# An Integrated Electrochemical System for Synergistic Cathodic Nitrate Reduction and Anodic Sulfite Oxidation

**DOI:** 10.3390/molecules28124666

**Published:** 2023-06-09

**Authors:** Bing Cui, Shizhao Wang, Xiaofu Guo, Yingying Zhao, Sohrab Rohani

**Affiliations:** 1 Tianjin Key Laboratory of Chemical Process Safety, Hebei Collaborative Innovation Center of Modern Marine Chemical Technology, Engineering Research Center of Seawater Utilization of Ministry of Education, School of Chemical Engineering and Technology, Hebei University of Technology, Tianjin 300130, China; cuibing23412@163.com (B.C.); shizhaow@163.com (S.W.); guoxiaofu@hebut.edu.cn (X.G.); 2 Department of Chemical and Biochemical Engineering, Western University, London, ON N6A 5B9, Canada

**Keywords:** electrochemical, integrated system, nitrate reduction, sulfite oxidation

## Abstract

Electrochemical reduction of nitrate has broad application prospects. However, in traditional electrochemical reduction of nitrate, the low value of oxygen produced by the anodic oxygen evolution reaction and the high overpotential limit its application. Seeking a more valuable and faster anodic reaction to form a cathode–anode integrated system with nitrate reaction can effectively accelerate the reaction rate of the cathode and anode, and improve the utilization of electrical energy. Sulfite, as a pollutant after wet desulfurization, has faster reaction kinetics in its oxidation reaction compared to the oxygen evolution reaction. Therefore, this study proposes an integrated cathodic nitrate reduction and anodic sulfite oxidation system. The effect of operating parameters (cathode potential, initial NO_3_^−^–N concentration, and initial SO_3_^2−^–S concentration) on the integrated system was studied. Under the optimal operating parameters, the nitrate reduction rate in the integrated system reached 93.26% within 1 h, and the sulfite oxidation rate reached 94.64%. Compared with the nitrate reduction rate (91.26%) and sulfite oxidation rate (53.33%) in the separate system, the integrated system had a significant synergistic effect. This work provides a reference for solving nitrate and sulfite pollution, and promotes the application and development of electrochemical cathode–anode integrated technology.

## 1. Introduction

In recent years, accelerated industrialization and increasing human activities have led to severe nitrate pollution [1]. Compared with other techniques, such as biological and adsorption methods, the electrochemical reduction of nitrate technology has the advantages of fast removal rate, low cost, and no chemical additives [2,3,4]. It can directly convert harmful nitrate into valuable ammonia, making it a promising nitrate treatment technology. In the traditional electrochemical reduction of nitrate process, the anodic half-reaction is the oxygen evolution reaction (OER), and the economic value of oxygen is limited, so it is usually directly released into the air. In addition, the occurrence of OER requires a high overpotential, which to some extent limits the nitrate reduction rate and causes energy waste [5]. Up to now, researchers have always been keen to develop new materials to obtain higher nitrate reduction rates and product selectivity in exploring the electrochemical reduction of nitrate, and there is almost no research focusing on the application of anodic reactions.

Seeking more valuable anodic reactions to match with nitrate reduction reactions to form an integrated cathodic reduction–anodic oxidation system is highly desirable. In contrast to a single anode or cathode reaction, the integrated cathode reduction–anode oxidation system simultaneously focuses on the anode and cathode reactions, achieving matching reaction rates in a unit system [6]. As a result, the electrochemical cathode–anode integrated system has significant advantages: (1) the matching reaction between the cathode and anode synergistically improves the reaction rates; (2) integrated cathode–anode can simultaneously treat two systems, significantly reducing process costs; and (3) cathode–anode coupling replaces the hydrogen evolution reaction (HER) and oxygen evolution reaction (OER) in separate oxidation and reduction processes, improving energy utilization efficiency [7,8,9].

Sulfite is the main product after wet desulfurization; excess sulfite will cause water and soil pollution, decomposition will release SO_2_ again, and timely oxidation of sulfite to sulfate can effectively avoid secondary pollution [10,11]. Compared to OER, the oxidation reaction of sulfite (SOR) generated in the desulfurization process exhibits faster kinetics [9,12]. Therefore, by combining cathodic nitrate reduction with anodic sulfite oxidation through electrochemical reactions, synergistic efficiency can be achieved in treating wastewater containing nitrate and sulfite.

In this study, the anodic sulfite oxidation reaction (SOR) was employed to replace the OER, and an electrochemical system integrating cathodic NO_3_^−^RR with anodic SOR was constructed for the synergistic treatment of both wastewaters. The optimal treatment conditions of the coupled system were determined by investigating the influencing factors including the cathodic potential, initial nitrate concentration, and initial sulfite concentration. The development of this integrated system could provide valuable insights into the development and applications of synergistic electrochemical technology.

## 2. Results and Discussion

### 2.1. Characterizations of CuO&Cu_2_O@C Electrode

**Figure 1:**
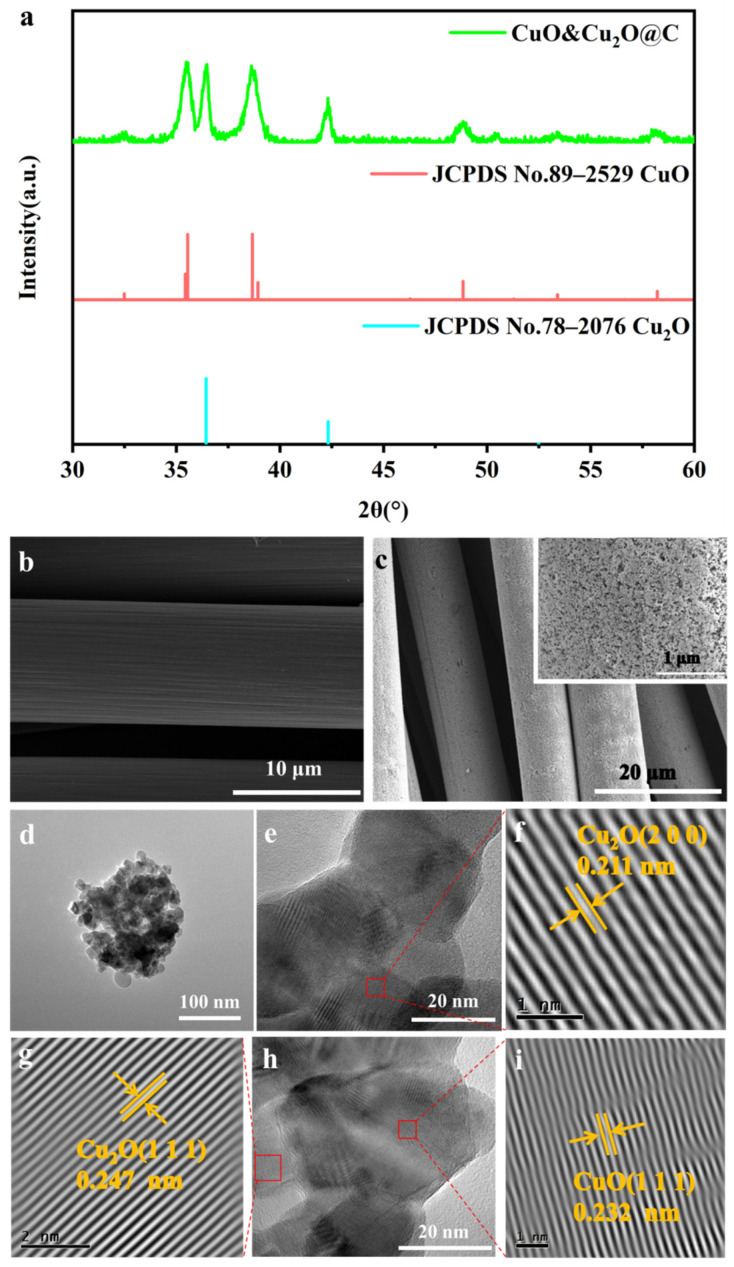
(**a**) XRD patterns of CuO&Cu_2_O@C; (**b**) SEM image of CC; (**c**) SEM images of CuO&Cu_2_O@C; (**d**) TEM image; (**e**–**i**) HRTEM images of CuO&Cu_2_O@C.

The physical phases of the constructed electrode were analyzed via XRD. Figure 1a shows the electrode as a mixed composition of CuO (JCPDS No. 78–2076) and Cu_2_O (JCPDS No. 89–2529). The strong diffraction peaks at 36.4° and 42.3° are attributed to the (1 1 1) and (2 0 0) crystal planes of Cu_2_O. The characteristic diffraction peaks at 35.6°, 38.7°, 48.8°, and 58.2° are associated with the (−1 1 1) (1 1 1) (−2 0 2) (2 0 0) crystalline plane of CuO. SEM characterization was used to analyze the surface morphology of the prepared electrode. As shown in Figure 1b,c, the surface of the CC substrate material was uncontaminated, and the constructed electrode surface formed uniform nanoparticles. In order to further clarify the composition and microstructure of the surface nanoparticles, TEM characterization was performed. The TEM image in Figure 1d further shows that nanoparticles were forming on the CC surface. In the HRTEM image (Figure 1e–i), the lattice spacing of the stripes with 0.211 nm, 0.247 nm, and 0.232 nm is attributed to the (2 0 0) and (1 1 1) crystal faces of Cu_2_O and the (1 1 1) crystal face of CuO [13,14], respectively. This is consistent with the XRD results and fully demonstrates that the composition of the nanoparticles formed on the electrode surface is CuO and Cu_2_O.

**Figure 2:**
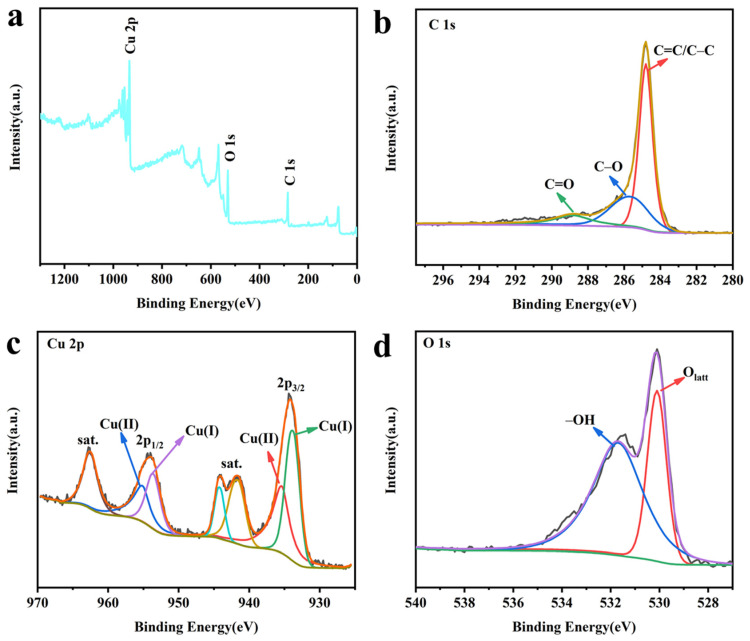
(**a**) XPS full-range spectra; (**b**) high-resolution Cu 1s; (**c**) high-resolution Cu 2p; (**d**) high-resolution O 1s of CuO&Cu_2_O@C.

In order to gain a deeper understanding of the nanoparticles on the electrode surface, the element composition and related valence states of the electrode were analyzed via XPS characterization. The XPS full spectrum (Figure 2a) shows the presence of Cu and O elements on the electrode surface, further proving the existence of Cu–related species on the electrode surface. As shown in Figure 2b, the high-resolution C1s spectrum was deconvoluted into three peaks, which could be assigned as C=C/C–C (284.8 eV), C–O (285.6 eV), and C=O (288.4 eV), respectively [13]. The high-resolution Cu 2p spectrum (Figure 2c) contains characteristic peaks of Cu(I) and Cu(II). The peaks at 933.8 eV and 953.6 eV are attributed to the Cu 2p_3/2_ and Cu 2p_1/2_ of Cu_2_O, respectively [15]. A clear satellite peak appears near the peaks at 935.4 eV and 955.1 eV, which is a typical feature of Cu(II) [16]. The high-resolution O 1s spectra (Figure 2d) can be deconvoluted into two peaks, which are hydroxyl oxygen (–OH, 531.7 eV) and lattice oxygen (O_latt_, 530.1 eV) [17]. The XPS results are consistent with the TEM results, further proving the synthesis of the CuO&Cu_2_O@C electrode.

### 2.2. Electrochemical Reduction of Nitrate

**Figure 3:**
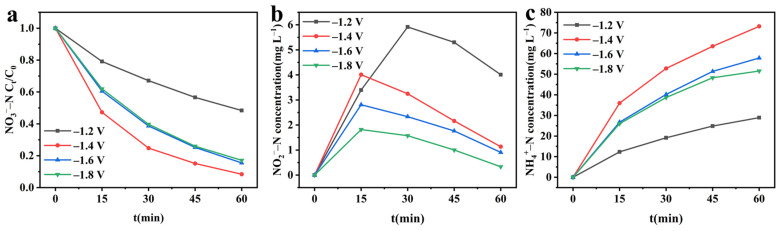
Electrochemical reduction of nitrate at different cathodic potentials. (**a**) NO_3_^−^–N concentration; (**b**) NO_2_^−^–N concentration; (**c**) NH_4_^+^–N concentration.

After confirming the structure of the CuO&Cu_2_O@C electrode, the nitrate reduction activity of this electrode was investigated using different cathodic potentials. Figure 3a shows a significant decay trend of nitrate concentration within 60 min under different potentials, indicating that CuO/Cu_2_O@C electrode can effectively reduce nitrate. As the potential increases from −1.2 V to −1.8 V, the removal rate shows a volcano-like change. When applying −1.2 V cathodic potential, the removal rate is the lowest, at only 51.59%. This is because at a lower potential, there are not enough electrons provided to reduce nitrate [18]. As the cathodic potential further increases to −1.4 V, the nitrate removal rate increases to 91.61%. This is because the increase in the cathodic potential results in an increase in the amount of electron transfer in the system. When the cathodic potential becomes more negative than −1.4 V, the competitive hydrogen evolution reaction gradually increases, leading to the inhibition of nitrate reduction reaction and a gradual decrease in the removal rate [19]. In order to evaluate the rate of electrochemical reduction of nitrate, the nitrate reduction process was fitted with a pseudo-first-order kinetic model, and the result is shown in Figure S1. R^2^ indicates that the nitrate reduction conforms to the pseudo-first-order kinetic model and has the fastest reaction rate at −1.4 V, with a kinetic constant (k_r_) of 2.4386 h^−1^.

To investigate the reduction products of nitrate, the concentration changes of NO_2_^−^–N and NH_4_^+^–N were continuously detected. In the early stage of the reaction, the concentrations of both NO_2_^−^–N and NH_4_^+^–N showed an increasing trend. However, in the later stages of the reaction, the concentration of NO_2_^−^–N showed a decreasing trend, while NH_4_^+^–N continued to increase (Figure 3b,c). This indicates that NO_2_^−^–N is the intermediate product of NO_3_^−^–N reduction, and NH_4_^+^–N is the final product of nitrate reduction [20]. The cumulative amount of NH_4_^+^–N increases with the increase of cathodic potential, which is consistent with the change law of nitrate removal rate, and also shows a volcano-like shape. This is because the decay of nitrate determines the generation of the final product NH_4_^+^–N. At high nitrate removal rates, it allows more NO_3_^−^–N to be converted to NH_4_^+^–N.

### 2.3. Self-Oxidation and Anodic Electro-Oxidation of Sulfite

**Figure 4:**
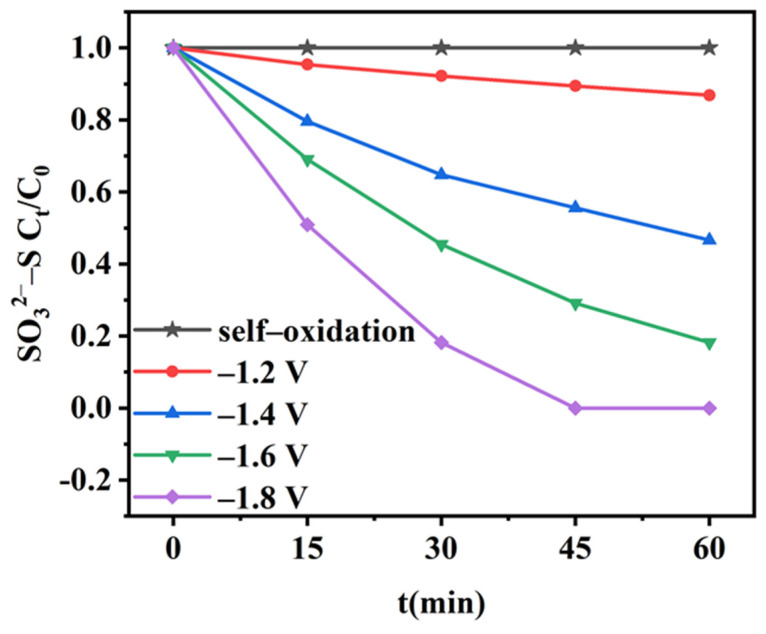
SO_3_^2−^–S concentration during self-oxidation and electro-oxidation at different potentials.

To investigate the electro-oxidation performance of SO_3_^2−^–S on a Pt sheet electrode, a separate study of the electrochemical oxidation of sulfite was conducted, and self-oxidation experiments were conducted to eliminate the effect of air on sulfite oxidation. As shown in Figure 4, the SO_3_^2−^–S concentration remained essentially constant during the self-oxidation, indicating that SO_3_^2−^–S was not oxidized by air in the H-type sealed electrolytic cell. In addition, the oxidation of SO_3_^2−^–S was all electrically driven during the electro-oxidation process. As the cathodic potential increased from −1.2 V to −1.8 V, the oxidation rate of SO_3_^2−^–S increased from 13.5% to 100%; moreover, the oxidation was complete within 45 min at −1.8 V. This demonstrates that the Pt electrode possessed effective SO_3_^2−^–S oxidization capability. The electro-oxidation process was fitted with a pseudo-first-order kinetic model, and the kinetic constants obtained for different cathodic potentials indicated that the electro-oxidation process of SO_3_^2−^–S conforms to the pseudo-first-order kinetic process (Figure S2). As the cathodic potential increases, the kinetic constant of the electro-oxidation process of SO_3_^2−^–S also increases, which is because at higher potentials, more electrons are generated, thereby accelerating the reaction rate [21].

### 2.4. Integrated Electrochemical System

Based on the single system of electrochemical reduction of nitrate and sulfite oxidation, the two were paired to construct an integrated system to achieve simultaneous treatment of nitrate and sulfite.

#### 2.4.1. Effect of the Applied Cathode Potentials

**Figure 5:**
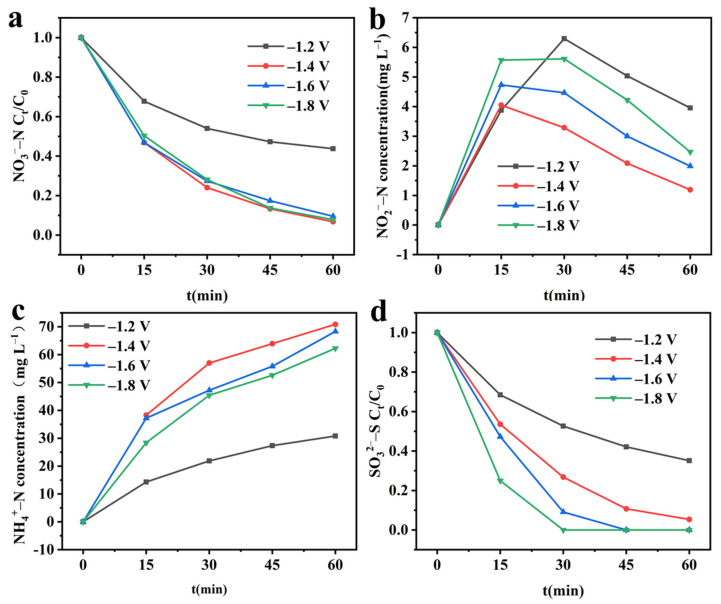
Effect of cathodic potential on the performance of integrated electrochemical systems. (**a**) NO_3_^−^–N concentration; (**b**) NO_2_^−^–N concentration; (**c**) NH_4_^+^–N concentration; (**d**) SO_3_^2−^–S concentration. Experimental conditions: initial NO_3_^−^–N concentration = 100 mg L^−1^; initial SO_3_^2−^–S concentration = 1.0 g L^−1^.

In electrochemical reactions, electrode potential plays a crucial role; therefore, the effect of cathode potential on the integrated system was explored. As shown in Figure 5a, in the integrated system, as the cathode potential increases from −1.2 V to −1.4 V, the NO_3_^−^–N removal rate increases from 56.28% to 93.26%. Continuing to improve the cathode potential to −1.8 V, the removal rate remains above 90%. The pseudo-first-order kinetic model fitting results for NO_3_^−^–N reduction (Figure S3a) indicate that NO_3_^−^–N reduction in the integrated system still belongs to the pseudo-first-order kinetic process. The reaction kinetic constant is highest at a cathode potential of −1.4 V, reaching 2.6622 h^−1^. Figure 5b,c shows the changes in the products NO_2_^−^–N and NH_4_^+^–N in the integrated system at different potentials. The accumulation of NO_2_^−^–N remains at a low level of 5 mg L^−1^ for 1 h at different potentials. The accumulation of NH_4_^+^–N increases first and then decreases with the increase of potential, and the highest accumulation was −1.4 V. The reason for the above phenomenon is consistent with that in the single reduction system. At lower potentials, the insufficient electrons produced eventuate NO_3_^−^–N removal, while at higher potentials, a large amount of hydrogen evolution reaction becomes the primary reaction, hindering the conversion of NO_3_^−^–N [22]. As shown in Figure 5d, with the increase of cathode potential, the oxidation rate of SO_3_^2−^–S in the integrated system increases from 64.91% to 100%. As shown in Figure S3b, the oxidation of SO_3_^2−^–S in the integrated system is also a pseudo-first-order kinetic process.

Table 1 shows the comparison of performance parameters between single electrochemical systems and integrated electrochemical systems. In the integrated system, the removal rate of NO_3_^−^–N and the oxidation rate of SO_3_^2−^–S are both improved at different cathode potentials. This implies that the constructed integrated system has synergistic effects. This is because in the single NO_3_^−^–N reduction system, the anode reaction is oxygen evolution, while in the integrated system, the anode reaction is SO_3_^2−^–S oxidation, which has lower activation energy and only requires two electrons to complete the reaction, while the oxygen evolution reaction has higher activation energy and requires four electrons. As a result, the oxidation reaction rate SO_3_^2−^–S is faster in the integrated system, making for a higher reaction current, which promotes the reduction of NO_3_^−^–N [12]. As for the single sulfite reaction system, the cathode is hydrogen evolution reaction, and the hydrogen evolution reaction rate of the prepared CuO&Cu_2_O@C electrode is slow. However, in the integrated system, the CuO&Cu_2_O@C electrode accelerates the reduction rate of NO_3_^−^–N, and thus elevates the ability of the anode to oxidize SO_3_^2−^–S. It is worth noting that the enhancement amplitude of NO_3_^−^–N removal is low at lower potentials of −1.2 V and −1.4 V, while it is significant at higher potentials of −1.6 V and −1.8 V. This is because at high negative potentials, a large amount of hydrogen evolution reaction occurs in the single reduction system, which inhibits the reduction of NO_3_^−^–N, while at low potentials, the hydrogen evolution reaction does not occur violently [23]. In the integrated system, SO_3_^2−^–S oxidation inhibits the occurrence of hydrogen evolution reaction [24], and therefore the magnitude of the potentiation varies at different potentials. In addition, the increase of NH_4_^+^–N selectivity at different potentials is consistent with the pattern of NO_3_^−^RR increase. This is due to the fact that NO_3_^−^RR is directly related to the formation of the final product NH_4_^+^–N [25].

By comparing various performance indicators, it can be seen that the NO_3_^−^–N removal rate is optimal at −1.4 V, the NO_2_^−^–N selectivity is the lowest, the NH_4_^+^–N selectivity is the highest, and although the SO_3_^2−^–S oxidation rate is not the highest, it still exhibits excellent performance. Therefore, it was reasonable to choose −1.4 V for the subsequent experiments.

#### 2.4.2. Effect of Initial NO_3_^−^–N Concentration

**Figure 6:**
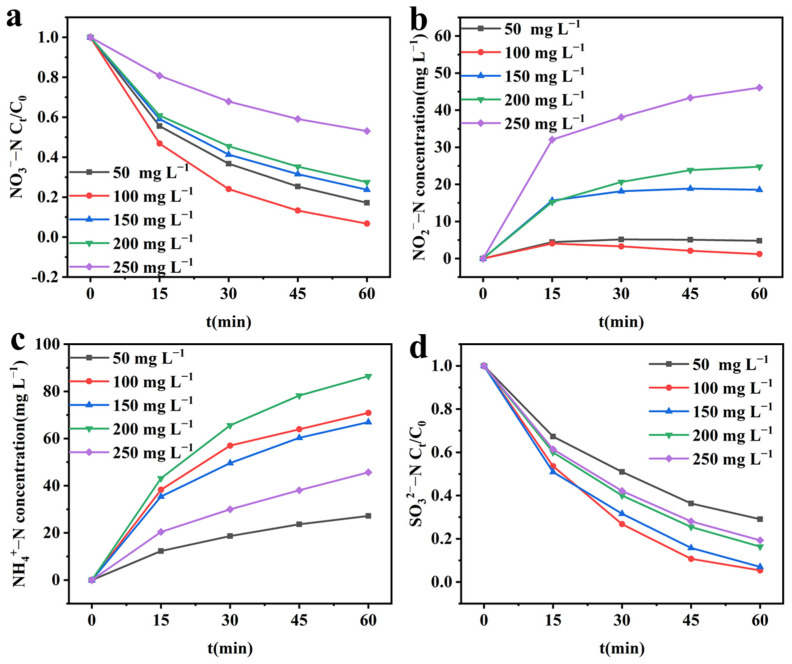
Effect of initial NO_3_^−^–N concentration on the performance of integrated electrochemical systems. (**a**) NO_3_^−^–N concentration; (**b**) NO_2_^−^–N concentration; (**c**) NH_4_^+^–N concentration; (**d**) SO_3_^2−^–S concentration. Experimental conditions: cathodic potential = −1.4 V; initial SO_3_^2−^–S concentration = 1.0 g L^−1^.

In integrated electrochemical systems, changes in the half-reaction rate can affect the overall system reaction rate [26]. Changes in the initial NO_3_^−^–N concentration affect the cathodic reaction NO_3_^−^RR rate [27], so the effect of different initial NO_3_^−^–N concentrations on the integrated system was investigated. As shown in Figure 6a and Figure S4a, as the initial NO_3_^−^–N concentration increases from 50 mg L^−1^ to 100 mg L^−1^, the NO_3_^−^–N removal rate first increases and reaches a maximum of 93.26% at 100 mg L^−1^, with the fastest reaction rate. As the initial NO_3_^−^–N concentration continues to increase, the NO_3_^−^–N removal rate continues to decrease, with the lowest removal rate at 250 mg L^−1^, only 46.91%, and the slowest reaction rate. The lower removal rate at a low initial NO_3_^−^–N concentration is due to the limited binding capacity of NO_3_^−^–N, which leads to a decrease in the effective collision frequency of active molecules [28]. At higher concentrations, the lower NO_3_^−^–N removal rate is due to the limited catalytic active sites on the CuO&Cu_2_O@C electrode surface [29]. As shown in Figure 6b,c, with the increase of initial NO_3_^−^–N concentration, the selectivity of NO_2_^−^–N first increases and then decreases, while the selectivity of NH_4_^+^–N shows the opposite trend. This is because at low initial NO_3_^−^–N concentrations, the rate of conversion of NO_3_^−^–N to the intermediate product NO_2_^−^–N is lower than the rate of conversion of NO_2_^−^–N to NH_4_^+^–N, while at higher initial NO_3_^−^–N concentrations, the rate of conversion of NO_3_^−^–N to the intermediate product NO_2_^−^–N is higher than the rate of conversion of NO_2_^−^–N to NH_4_^+^–N.

As shown in Figure 6d, as the initial NO_3_^−^–N concentration increases from 50 mg L^−1^ to 100 mg L^−1^, the SO_3_^2−^–S oxidation rate increases from 70.91% to 94.64%. As the initial NO_3_^−^–N concentration further increases, the SO_3_^2−^–S oxidation rate gradually decreases and decreases to 80.70% at 250 mg L^−1^. As shown in Figure S4b, the oxidation kinetic constant k_o_ first increases and then decreases with the rise in initial NO_3_^−^–N concentration, which is consistent with the changing trend of cathodic reduction NO_3_^−^–N kinetic constant. This indicates that the change in the cathodic reaction rate will cause the same change trend in the anodic reaction rate, which is due to the mutual matching relationship between the cathodic and anodic reaction rates. When the rates of the two half-reactions do not match, they will decrease simultaneously.

By comparing the NO_3_^−^–N reduction rate, SO_3_^2−^–S oxidation rate, NO_2_^−^–N selectivity, and NH_4_^+^–N selectivity, it is easy to find that the initial NO_3_^−^–N concentration of 100 mg L^−1^ showed excellent performance in all the above indexes, so it was chosen as the initial NO_3_^−^–N concentration for the subsequent study.

#### 2.4.3. Effect of Initial SO_3_^2−^–S Concentration on Integrated Electrochemical System

**Figure 7:**
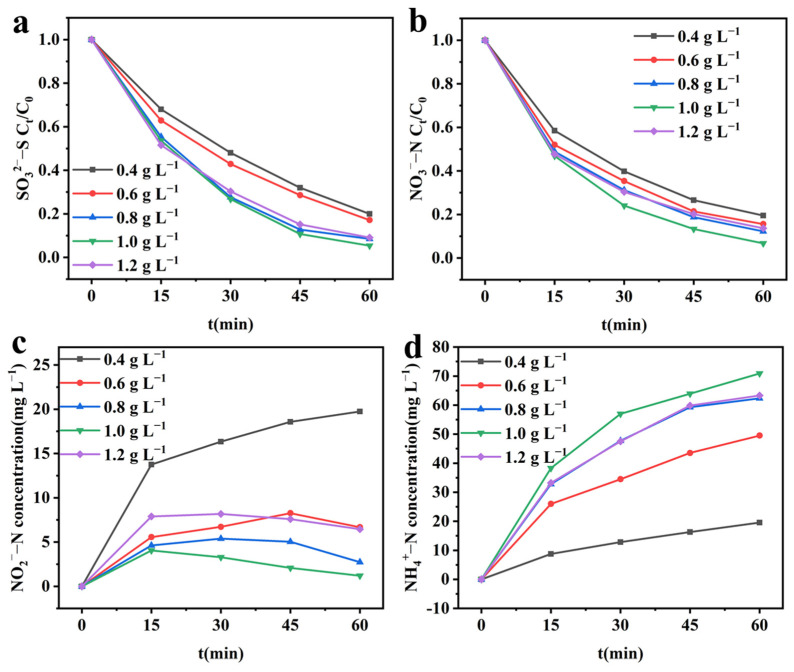
Effect of initial SO_3_^2−^–S concentration on the performance of integrated electrochemical systems. (**a**) SO_3_^2−^–S concentration; (**b**) NO_3_^−^–N concentration; (**c**) NO_2_^−^–N concentration; (**d**) NH_4_^+^–N concentration. Experimental conditions: cathodic potential = −1.4 V; initial NO_3_^−^–N concentration = 100 mg L^−1^.

The initial SO_3_^2−^–S concentration in the anode chamber is closely related to the SOR rate [30], which affects the cathodic reaction rate. Based on this, the effect of initial SO_3_^2−^–S concentration on the integrated system was investigated. As shown in Figure 7a, as the initial SO_3_^2−^–S concentration increases from 0.4 g L^−1^ to 1.0 g L^−1^, the SO_3_^2−^–S oxidation rate increases continuously from 80.00% to 94.64%. When the initial SO_3_^2−^–S concentration is further increased to 1.2 g L^−1^, the SO_3_^2−^–S oxidation rate decreases to 90.91%. As the initial SO_3_^2−^–S concentration increases, the oxidation kinetic constant k_o_ shows a trend of first increasing and then declining (Figure S5a). At concentrations of 0.4, 0.6, 0.8, 1.0, and 1.2 g L^−1^, the reduction rates of NO_3_^−^–N are 80.51%, 84.42%, 87.80%, 93.26%, and 86.37%, respectively (Figure 7b). The fitting results of cathodic reduction NO_3_^−^–N to first-order kinetics show (Figure S5b) that as the initial SO_3_^2−^–S concentration continuously increases, k_r_ increases from 1.5891 h^−1^ to 2.6622 h^−1^ and then decreases to 1.9384 h^−1^. This changing trend is consistent with the SO_3_^2−^-S oxidation kinetic constant trend. This once again indicates that at lower initial SO_3_^2−^–S concentration, the anodic oxidation reaction cannot match the cathodic reduction reaction, resulting in a decrease in the cathodic and anodic reaction rates in the integrated system. As the initial SO_3_^2−^–S concentration increases, the anodic and cathodic reactions gradually match, resulting in the highest rates of sulfite oxidation and nitrate reduction at an initial SO_3_^2−^–S concentration of 1.0 g L^−1^. When the initial SO_3_^2−^–S concentration continues to increase to 1.2 g L^−1^, this mismatch reappears, resulting in a decrease in the performance of the integrated system.

Figure 7c,d shows the changes of the products NO_2_^−^–N and NH_4_^+^–N at different initial SO_3_^2−^–S concentrations, respectively. The accumulation of NO_2_^−^–N first decreased with the increase of initial SO_3_^2−^–S concentration, reaching a minimum at 1.0 g L^−1^, and then increased with the further increase in SO_3_^2−^–S concentrations. The NH_4_^+^–N production showed a trend of increasing and then decreasing, with the highest production at the initial SO_3_^2−^–S concentration of 1.0 g L^−1^. This indicates that the decrease in the reaction rate during the cathodic reduction of nitrate leads to the accumulation of intermediate products, affecting the transformation of intermediate products to final products.

In this system, NO_3_^−^ is converted to NH_4_^+^ at the cathode and SO_3_^2−^ is converted to SO_4_^2−^ at the anode. The final products could be recycled via crystallization and utilized to form valuable compounds such as magnesium ammonia phosphate (struvite precipitate, NH_4_MgPO_4_·6H_2_O) [31,32], calcium sulfate (CaSO_4_), barium sulfate (BaSO_4_), Ettringite (Ca_6_Al_2_(SO_4_)_3_(OH)_12_) [33], etc., thus achieving more effective treatment of nitrate and sulfite in wastewater.

## 3. Materials and Methods

### 3.1. Reagents

The reagents in this work are of analytical grade or above and were used without further purification.

### 3.2. Fabrication of CuO&Cu_2_O@C Electrode

In this study, a Pt foil (1 cm × 1.5 cm × 0.1 mm) was used as the anode, and a Ag/AgCl electrode was used as the reference electrode. To investigate the accelerating effect of the SOR instead of OER, a highly NO_3_^−^RR-efficient CuO&Cu_2_O@C electrode (effective area of 1 cm × 1.5 cm) was synthesized and used as the cathode.

The CuO&Cu_2_O@C electrode was constructed using a redox strategy based on the previously reported research protocol with modifications [1,34]. Specifically, a carbon cloth (1 cm × 2 cm) was subjected to ultrasound treatment with a mixture of nitric acid (10 wt.% HNO_3_) and sulfuric acid (10 wt.% H_2_SO_4_), acetone, ethanol, and deionized water for 1 h to remove surface impurities. Subsequently, 8 mL of ethylene glycol, 8 mL of ethanol, and 3 mmol Cu(NO_3_)_2_·3H_2_O were added to a beaker, and the mixture was thoroughly stirred for 1 h to obtain a transparent blue solution. The pretreated carbon cloth was then immersed in the solution for 4 h, and the entire system was transferred to a 25 mL hydrothermal autoclave and reacted at 180 ℃ for 6 h. After the hydrothermal autoclave was cooled to room temperature, the modified carbon cloth was taken out and sonicated in anhydrous ethanol for 15 s, washed several times with water and ethanol, and dried at 60 ℃ in a vacuum oven for 6 h. Finally, the modified carbon cloth was annealed in a muffle furnace at 350 °C for 2 h to obtain the CuO&Cu_2_O@C electrode.

### 3.3. Characterizations

X-ray diffraction (XRD, Bruker, Billerica, MA, USA, D8 FOCUS) was used to analyze the physical phase of the electrode. Scanning electron microscopy (SEM, Zeiss, Oberkochen, Germany, G300) and transmission electron microscopy (TEM, FEI, Tecnai G2 F30) were used to record the morphology and crystal lattice information. X-ray photoelectron spectroscopy (XPS, Thermo, Waltham, MA, USA, ESCALAB 250XI, Al Kα) was applied to examine the chemical and binding states of the CuO&Cu_2_O@C electrode. The binding energy was calibrated using C 1s spectrum at 284.8 eV.

### 3.4. Batch Experiments

**Figure 8:**
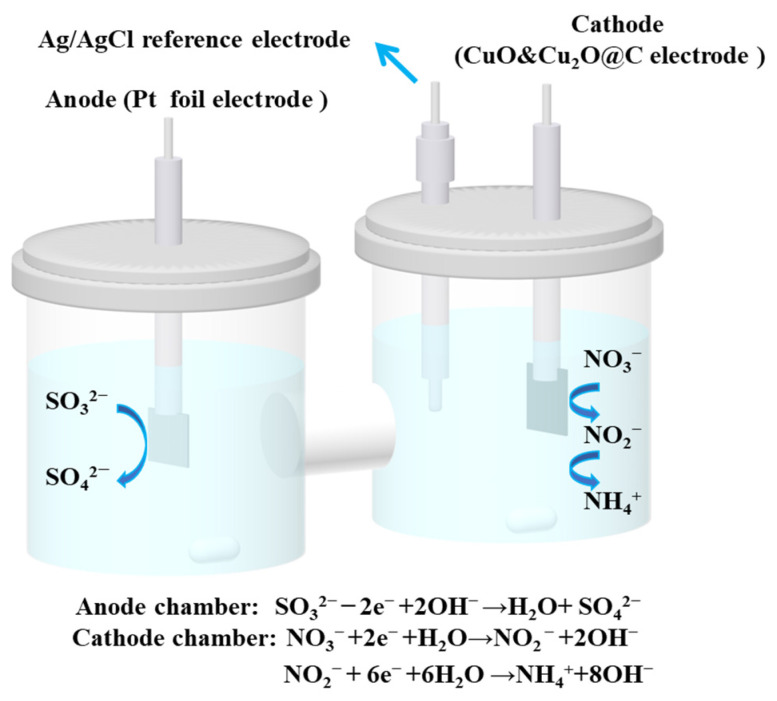
H-type electrolytic cell and SO_3_^2−^, NO_3_^−^ transformation process.

Batch experiments were conducted in an H-type electrolytic cell (Figure 8), with an anode and cathode distance of 8 cm, separated by a Nafion 117 proton exchange membrane, and using Ag/AgCl as the reference electrode.

#### 3.4.1. Single Electrochemical Reduction of Nitrate

In a three-electrode system, a mixture of 30 mL of 100 mg L^−1^ (K)NO_3_^−^–N and 0.5 mol L^−1^ K_2_SO_4_ solution was added to the cathode chamber, and 30 mL 0.5 mol L^−1^ K_2_SO_4_ solution was added to the anode chamber. The cathodic potentials of −1.2 V, −1.4 V, −1.6 V, and −1.8 V (vs. Ag/AgCl) were applied to drive the reactions to study the electrochemical reduction of nitrate systems alone.

#### 3.4.2. Self-Oxidation and Single Electro-Oxidation of Sulfite

In a three-electrode system, 30 mL of 0.5 mol L^−1^ K_2_SO_4_ solution was added to the cathode chamber, and a mixture of 30 mL of 1.0 g L^−1^ (K_2_)SO_3_^2−^–S and 0.5 mol L^−1^ K_2_SO_4_ solution was added to the anode chamber. The study on sulfite self-oxidation was conducted without applying an electric current. In the single electrochemical oxidation of sulfite, in order to investigate the oxidation rate in the absence of nitrate reduction reaction, the aforementioned potential was still used for the study.

#### 3.4.3. Integrated Electrochemical Batch Experiments

The cathode compartment was filled with a mixed solution of 30 mL of 1.0 g L^−1^ (K_2_)SO_3_^2−^–S and 100 mg L^−1^ (K)NO_3_^−^–N, while the anode compartment was filled with 30 mL of 1.0 g L^−1^ (K_2_)SO_3_^2−^–S and 0.5 mol L^−1^ K_2_SO_4_ mixed solution. Both the cathode and anode compartments were stirred at 300 rpm, and the batch experiments were conducted for 1 h. At a time interval of 15 min, 0.4 mL of solution was taken from the cathode compartment to detect the concentrations of NO_3_^−^–N, NO_2_^−^–N, and NH_4_^+^–N, and 1 mL of solution was taken from the anode compartment to detect the concentration of SO_3_^2−^–S. The method for the determination of ion concentrations and calculation for removal rate and selectivity were illustrated in the supporting information. Additionally, this study investigated the effects of different cathode potentials (−1.2 V, −1.4 V, −1.6 V, −1.8 V vs. Ag/AgCl), initial NO_3_^−^–N concentration (50 mg L^−1^, 100 mg L^−1^, 150 mg L^−1^, 200 mg L^−1^, 250 mg L^−1^), and initial SO_3_^2−^–S concentration (0.4 g L^−1^, 0.6 g L^−1^, 0.8 g L^−1^, 1.0 g L^−1^, 1.2 g L^−1^) on the integrated system.

## 4. Conclusions

In summary, a cathode material for efficient electrochemical reduction of nitrate was designed, which achieved effective removal of nitrate. However, in the single reduction process, the anodic oxygen evolution reaction (OER) has certain limitations and wastes energy. Therefore, an electrochemical reduction of nitrate integrated with a sulfite oxidation system was developed. By exploring the operating parameters (cathode potential, initial nitrate concentration, and initial sulfite concentration) in the integrated system, the results show that compared with the single reduction and oxidation system, the integrated system has a faster reaction rate while achieving simultaneous treatment of harmful nitrate and sulfite. At −1.4 V (vs. Ag/AgCl) cathodic potential, 100 mg L^−1^ initial nitrate, and 1.0 g L^−1^ sulfite concentrations, the nitrate removal rate and sulfite oxidation rate within 1 h reached 93.26% and 94.64%, respectively. This study provides more references for solving the mutual constraint between half-reactions.

## Figures and Tables

**Table 1:** Comparison of single system and integrated system performance parameters.

	*R* (NO_3_^−^–N)/%		*S* (NO_2_^−^–N)/%		*S* (NH_4_^+^–N)/%		*O* (SO_3_^2−^–S)/%	
	Single	Integrated	Single	Integrated	Single	Integrate	Single	Integrated
−1.2 V	51.59	56.28	7.64	6.80	55.14	53.50	13.15	64.91
−1.4 V	91.61	93.26	1.22	1.25	78.41	74.90	53.33	94.64
−1.6 V	84.46	90.46	1.05	2.10	67.21	73.20	81.82	100.00
−1.8 V	82.89	92.19	0.40	2.60	61.39	66.70	100.00	100.00

## Data Availability

The datasets used and analyzed in the present study are available from the corresponding author upon reasonable request.

## Supplementary Materials

The following supporting information can be downloaded at: https://www.mdpi.com/article/10.3390/molecules28124666/s1, Figure S1: Fitting results of pseudo-first-order kinetic model for single electrochemical reduction of nitrate at different potentials. Figure S2. Fitting results of pseudo-first-order kinetic model for single electrochemical oxidation of sulfite at different potentials. Figure S3. Fitting results of pseudo-first-order kinetic model for integrated electrochemical system at different potentials; (a) nitrate reduction reaction; (b) sulfite oxidation reaction. Figure S4. Fitting results of pseudo-first-order kinetic model for integrated electrochemical system at different initial NO_3_^−^–N concentrations; (a) nitrate reduction reaction; (b) sulfite oxidation reaction. Figure S5. Fitting results of pseudo-first-order kinetic model for integrated electrochemical system at different initial SO_3_^2−^–S concentrations; (a) sulfite oxidation reaction; (b) nitrate reduction reaction.

## Sample Availability

Samples of the compounds are available from the authors.
