# Supplementary material for: An Integrated Electrochemical System for Synergistic Cathodic Nitrate Reduction and Anodic Sulfite Oxidation
Source: Molecules. 2023 Jun 9;28(12):4666. doi: 10.3390/molecules28124666 (PMC10302429; doi:10.3390/molecules28124666)
Supplement: Supplementary file 1 [file molecules-28-04666-s001.zip › molecules-2428655-supplementary.pdf]

Supporting Information

An Integrated Electrochemical System for Synergistic Cathodic Nitrate Reduction and Anodic Sulfite Oxidation

Bing Cui [1], Shizhao Wang [1], Xiaofu Guo [1], Yingying Zhao [1,*] and Sohrab Rohani [2,*]

[1] Tianjin Key Laboratory of Chemical Process Safety, Hebei Collaborative Innovation Center of Modern Marine Chemical Technology, Engineering Research Center of Seawater Utilization of Ministry of Education, School of Chemical Engineering and Technology, Hebei University of Technology, Tianjin 300130, China; cuibing23412@163.com (B.C.); shizhaow@163.com (S.W.); guoxiaofu@hebut.edu.cn (X.G.)

[2] Department of Chemical and Biochemical Engineering, Western University, London, ON N6A 5B9, Canada

* Correspondence: luckyzhaoyy@126.com (Y.Z.); srohani@uwo.ca (S.R.)

S1. Analytical methods for NO_3^-RR

S1.1 Testing methods for the ions

The ionic testing methods for NO_3^-RR are same with the reported work.

S1.2 Calculation methods for relevant parameters

The removal efficiency of NO_3^-–N ($R(NO_3^-–N)$), selectivity of NO_2^-–N ($S(NO_2^-–N)$), selectivity of NH_4^+–N ($S(NH_4^+–N)$),can be calculated using **Eqs. S1-S3**.

$$R(NO_3^-–N)=\frac{C_0(NO_3^-–N)-C_t(NO_3^-–N)}{C_0(NO_3^-–N)}\times100\% \quad (S1)$$

$$S(NO_2^-–N)=\frac{C_t(NO_2^-–N)-C_0(NO_2^-–N)}{C_0(NO_3^-–N)-C_t(NO_3^-–N)}\times100\% \quad (S2)$$

$$S\left(NH_4^+–N\right)=\frac{C_t\left(NH_4^+–N\right)-C_0\left(NH_4^+–N\right)}{C_0(NO_3^-–N)-C_t(NO_3^-–N)}\times100\% \quad (S3)$$

where $C_0\left(NO_3^-–N\right)$, $C_0\left(NO_2^-–N\right)$ and $C_0\left(NH_4^+–N\right)$ are the initial concentrations of $NO_3^-–N$, $NO_2^-–N$, and $NH_4^+–N$, respectively, $C_t\left(NO_3^-–N\right)$, $C_t\left(NO_2^-–N\right)$ and $C_t\left(NH_4^+–N\right)$ are the concentrations of $NO_3^-–N$, $NO_2^-–N$, and $NH_4^+–N$ at time t, respectively,

Furthermore, the kinetics was fitted by pseudo-first order model (**Eq. 4**):

$$-\ln\left[\frac{C_t}{C_0}\right]=k_r t+A \quad (S4)$$

where k_r and A are fitting constants.

S2. Analytical methods for SOR

S2.1 Testing methods for the SO_3^{2-}

The SO_3^{2-} concentration was detected using the iodometric method (HG/T 2784-2012), which involved the following procedure:

(1) 5 mL of iodine standard solution was taken in an iodine flask and the volume

was marked as V_1. Then, 1 mL of the test solution containing SO_3^{2-} was added and mixed well;

(2) $Na_2S_2O_3$ solution was used to titrate the solution until it turned pale yellow, and then 2 mL of starch solution was added;

(3) The titration was continued with $Na_2S_2O_3$ solution until the blue color just faded away, and the volume of $Na_2S_2O_3$ solution consumed was recorded as V_2;

(4) A blank experiment was performed without the addition of the test solution containing sulfite ions, and the volume of $Na_2S_2O_3$ solution consumed was recorded as V_0.

S2.2 Calculation methods for relevant parameters

The concentration of SO_3^{2-} ($C(SO_3^{2-})$) and the oxidation rate ($O(SO_3^{2-})$) were calculated using **Eqs. S5-S6**.

$$C(SO_3^{2-}\text{-S})=\frac{16\times(V_0-V_2)\times C_{Na_2S_2O_3}}{V_1} \quad \text{(S5)}$$

$$O(SO_3^{2-}\text{-S})=\frac{C_0(SO_3^{2-}\text{–S})-C_t(SO_3^{2-}\text{–S})}{C_0(SO_3^{2-}\text{–S})} \quad \text{(S6)}$$

where $C_0(SO_3^{2-})$ and $C_t(SO_3^{2-})$ are the concentrations of SO_3^{2-}–S at time 0 and t, respectively, $C_{Na_2S_2O_3}$ represents the concentration of added $Na_2S_2O_3$ solution. The kinetics was also fitted by pseudo-first order model (k_o and B are fitting constants).

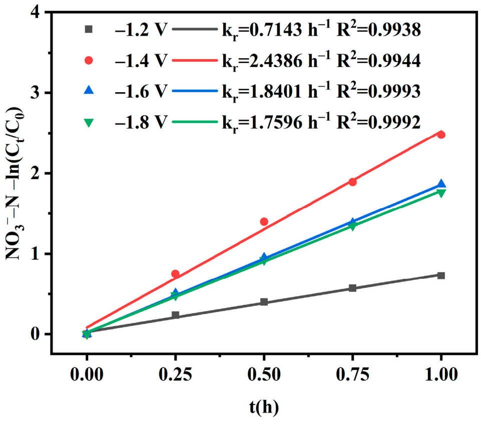

Figure S1. Fitting results of pseudo-first-order kinetic model for single electrochemical reduction of nitrate at different potentials.

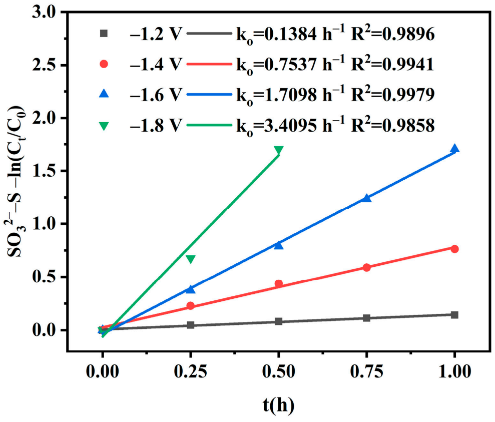

Figure S2. Fitting results of pseudo-first-order kinetic model for single electrochemical oxidation of sulfite at different potentials.

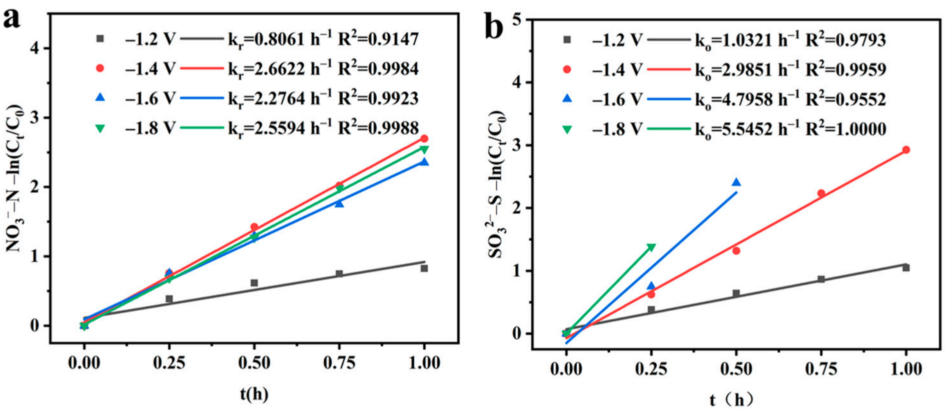

Figure S3. Fitting results of pseudo-first-order kinetic model for integrated electrochemical system at different potentials; (a) nitrate reduction reaction; (b) sulfite oxidation reaction.

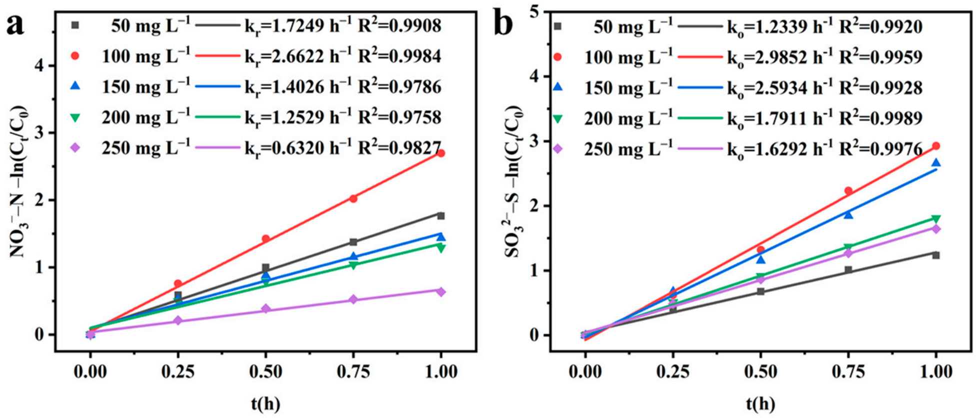

Figure S4. Fitting results of pseudo-first-order kinetic model for integrated electrochemical system at different initial NO_3^-–N concentrations; (a) nitrate reduction reaction; (b) sulfite oxidation reaction.

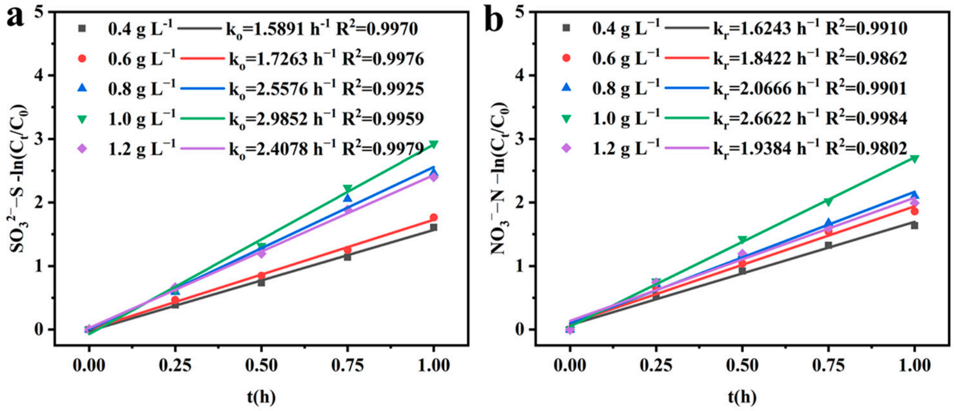

Figure S5. Fitting results of pseudo-first-order kinetic model for integrated electrochemical system at different initial SO_3^{2-}–S concentrations; (a) sulfite oxidation reaction; (b) nitrate reduction reaction.
